# Shelter availability and human attitudes as drivers of rock hyrax (*Procavia capensis*) expansion along a rural–urban gradient

**DOI:** 10.1002/ece3.6174

**Published:** 2020-04-15

**Authors:** Noam Ben‐Moshe, Takuya Iwamura

**Affiliations:** ^1^ School of Zoology Tel Aviv University Tel Aviv Israel

**Keywords:** invasion biology, *Leishmania tropica*, urban wildlife, zoonotic diseases

## Abstract

While anthropogenic land‐use changes threaten wildlife globally, some species take advantage of such changes and disperse into urban areas. The wildlife in urban areas often promotes conflicts with humans, notably when the animals are associated with the spread of zoonotic diseases. In Israel, current urban invasion of rock hyraxes (*Procavia capensis*) draws public attention, since the species is a reservoir host of cutaneous leishmaniasis, a serious skin disease. The rock hyrax, however, has seldom been studied in densely populated areas, and the drivers for its urban expansion, as well as its abilities to live and spread in core urban areas, are relatively unknown. Here, we explore the rock hyrax expansion to urban areas process by examining the availability, characteristics and use of shelter along an urban gradient. Our findings suggest that a series of factors determines shelter availability and quality for the rock hyrax, which facilitates its dispersion across the urban gradient. We found that rock hyraxes from the Judean Desert expand to the peri‐urban region of Jerusalem by colonizing new rocky shelters formed as by‐products of urban development. With their populations reaching extreme densities in this area and saturating the available shelters, there is some spill over to the adjacent core urban areas where they colonize littered sites, which are made available due to the local socio‐economic conditions and cultural norms of waste disposal and illegal placement of temporary structures. Our work emphasizes the significance of the urban gradient approach for studying the mechanisms promoting wildlife expansion to cities. Our findings suggest that changes in shelter availability and quality due to urban development, and cultural norms promote shifts of the hyrax population by pushing from the already established areas and pulling into new environment across the urban gradient.

## Graphical abstract

The rock hyrax (*Procavia capensis*) is expanding its range in Israel by colonizing artificial rock mounds formed by human development, sometimes near human settlements. This process has considerable implications on human health, as hyraxes are reservoir hosts of the leishmaniasis disease. In the article, we follow the process of the hyrax invasion to the city of Jerusalem along an urban gradient. We demonstrate how shelter availability and human attitudes facilitate the process, and the establishment of colonies in core urban areas.

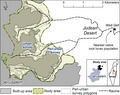

## 1 INTRODUCTION

Urbanization is one of the major globally drivers in reducing and fragmenting the land available to wild animals (Mcdonald, Kareiva, & Forman, 2008). Despite cumulative evidence of the negative impacts of urban development on wildlife species (Marzluff, 2002; Newbold et al., 2016), some wild animals have expanded their presence into urbanized areas and inhabit cities* *(Ditchkoff, Saalfeld, & Gibson, 2006; Lowry, Lill, & Wong, 2013). Newly colonized urban areas may compensate such animals for habitat loss in natural areas (Murphy, 1988; Rosenzweig, 2003; Soanes & Lentini, 2019), while the human residents may enjoy the wildlife locally (Dearborn & Kark, 2010; Marzluff, 2002). Urbanization also poses considerable risks to wild animals from predation or the transmission of diseases from domesticated animals (Gillies & Clout, 2003; Lepczyk, Mertig, & Liu, 2004), or roadkill (Forman & Alexander, 1998) and can negatively impact people's wellbeing through damage to property (Messmer, 2000), transmission of infectious zoonotic diseases (Daszak, Cunningham, & Hyatt, 2000; Patz et al., 2004), or physical attack (Soulsbury & White, 2015). Considering the increasing trends of urbanization, it is important to understand the drivers behind the urban expansion of wildlife in order to mitigate the negative impacts.

Wildlife expands to urban areas in a gradual procession along a rural‐to‐urban gradient as the levels of human development of an area create different ecological settings for the wild species (Evans, Hatchwell, Parnell, & Gaston, 2010; McKinney, 2008). Wildlife urban invasion may occur when animals are forced out of their native habitats through habitat loss (Markovchick‐Nicholls et al., 2008), overpopulation (Scott et al., 2001), or shortage of food or water (Davis, Taylor, & Major, 2011; Waite, Chhangani, Campbell, Rajpurohit, & Mohnot, 2007). They might be attracted to the urban environment by novel and unpopulated niches (Gahbauer et al., 2015), reliable food sources (Murray, Hill, Whyte, Cassady, & Clair, 2016), and reduced risk of predation (Gering & Blair, 1999; Rebolo‐Ifrán, Tella, & Carrete, 2017). Marzluff et al. (2001) found that intensive urbanization (i.e., urban core areas) negatively affect a wide range of both animal and plant species, while areas with mild urban development often have higher diversity than neighboring rural ones. Wildlife urban expansions along a full rural–urban gradient have been mostly studied in the context of the animals' occurrences (Birds and butterflies – Blair, 1999; mammalian carnivores ‐ Randa & Yunger, 2006; lizards – Germaine & Wakeling, 2001) or their behavior shifts along the gradient (Carrete & Tella, 2011Malach), but seldom through the changes in drivers that cause the expansion. To fully comprehend why wild animals leave their native habitats and settle in core urban areas, a comprehensive analysis of the changes in the drivers behind the expansion process along a rural–urban gradient is required.

**Figure 1:**
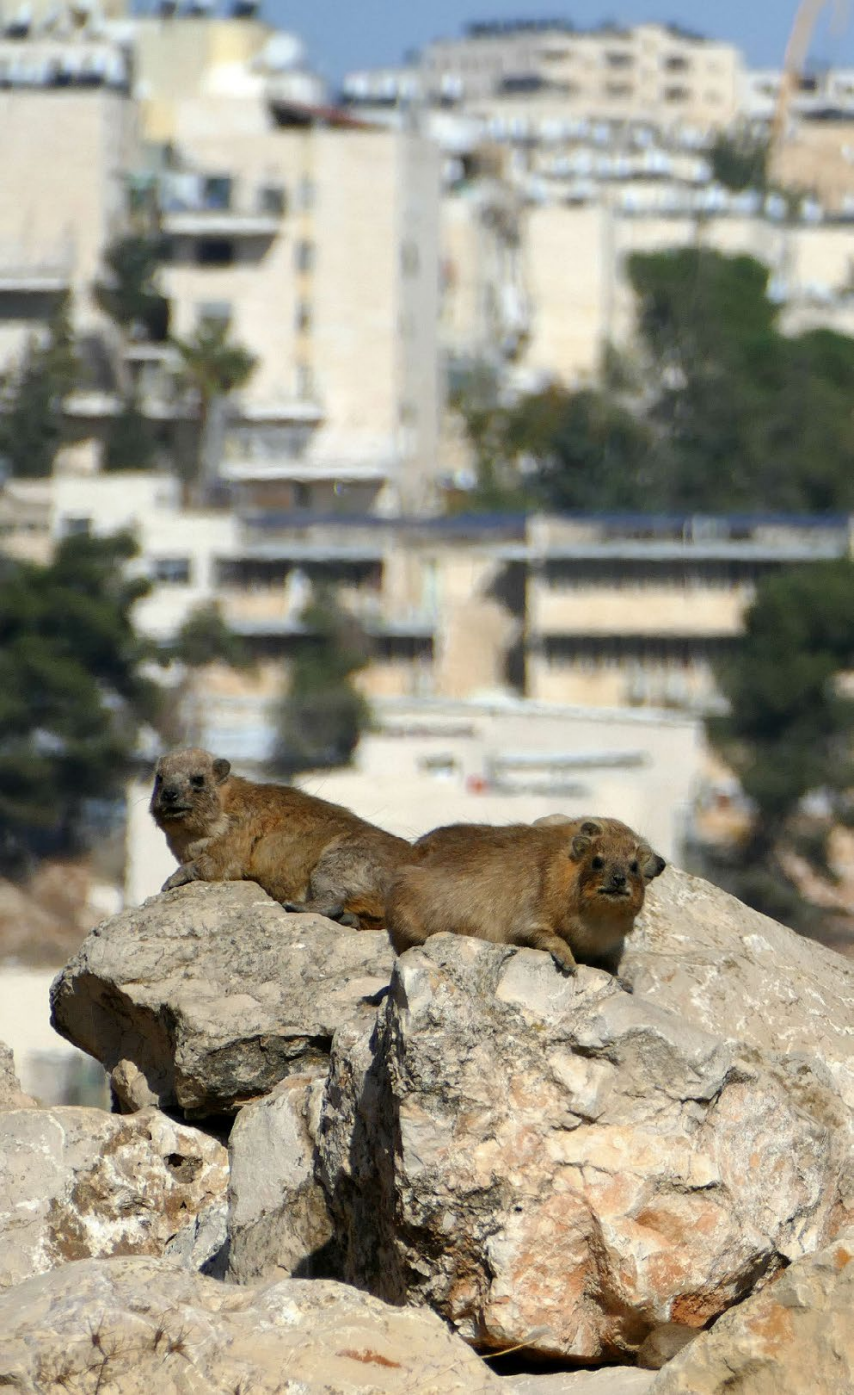
The rock hyrax, *Procavia capensis*, a medium‐sized (3–4 kg) social mammal found throughout the sub‐Sahara, North Africa and the Middle East. The species relies on rock piles used as shelters to escape from predators and extreme weather. In Israel, the hyraxes have expanded their distribution greatly by using artificial rock piles formed by human development and are settling near to human settlements. Their persistence next to humans is now considered as a health risk since they were found as reservoir hosts to leishmaniasis disease

Rock hyraxes (*Procavia capensis*) in Israel have emerged in the last 20 years at the outskirts of Jerusalem, a city with 930,000 residents (Israel Central Bureau of Statistics, 2019) (Figure 1). Historically, their distribution in Israel has been restricted to rocky landscapes (i.e., near cliffs and rock outcrops that they use as shelters; Meltzer & Livneh, 1982), but in the last 30 years they have expanded their range considerably due to an extensive increase in rock piles formed as by‐products of construction(Kershenbaum & Blaustein, 2011; Mendelssohn & Yom‐Tov, 1999). The hyrax expansion in Israel has created conflicts with humans through damage to crops and private gardens (Kershenbaum & Blaustein, 2011; Moran, Sofer, & Cohen, 1987) but particularly as a risk to public health. Rock hyraxes are considered as a main reservoir of *Leishmaina tropica*, a pathogenic protozoon causing the leishmaniaisis disease, which can be transmitted to humans by a sandfly sting (Talmi‐Frank et al., 2010). The rock hyrax emergence near Jerusalem and an outbreak of the disease in the city in 2013 (Solomon & Scwatrz, 2016) has hereby raised health concerns regarding their future expansion and ability to colonize highly populated urban areas. Previous research on rock hyraxes in South Africa has shown that they are able to inhabit human settlements, having developed s reduced fear of humans and learnt to exploit artificial shelters, and additional food sources near residential areas (Mbise et al., 2017; Naylor, 2015; Wiid & Butler, 2015). However, these and other previous studies of rock hyrax and human interactions (Kershenbaum & Blaustein, 2011; Moran et al., 1987) took place in natural or suburban environments and there is no documentation to date of rock hyraxes colonizing core urban areas. Naylor (2015), who studied the expansion of rock hyraxes into urban areas in South Africa, summarized that they occurred in suburbs but appeared to avoid densely urbanized areas.

Here, we used the urban gradient approach to explore the drivers behind the urban invasion of rock hyrax (*Procavia capensis*)* *from the Judean Desert into urban core areas in Jerusalem. We predicted that the drivers for such expansion would change with the level of urban development and exposure to humans. More specifically, we predicted that in peri‐urban areas these drivers would be based on the land cover changes associated with the urban development, while in urban area they would reflect human attitudes toward maintaining their living environment. Given the emergence of leishmaniasis in Jerusalem, we also predicted that the rock hyraxes would be found to already establish colonies inside the city (Figure 1).

## 2 METHODS

### 2.1 Study sites

**Figure 2:**
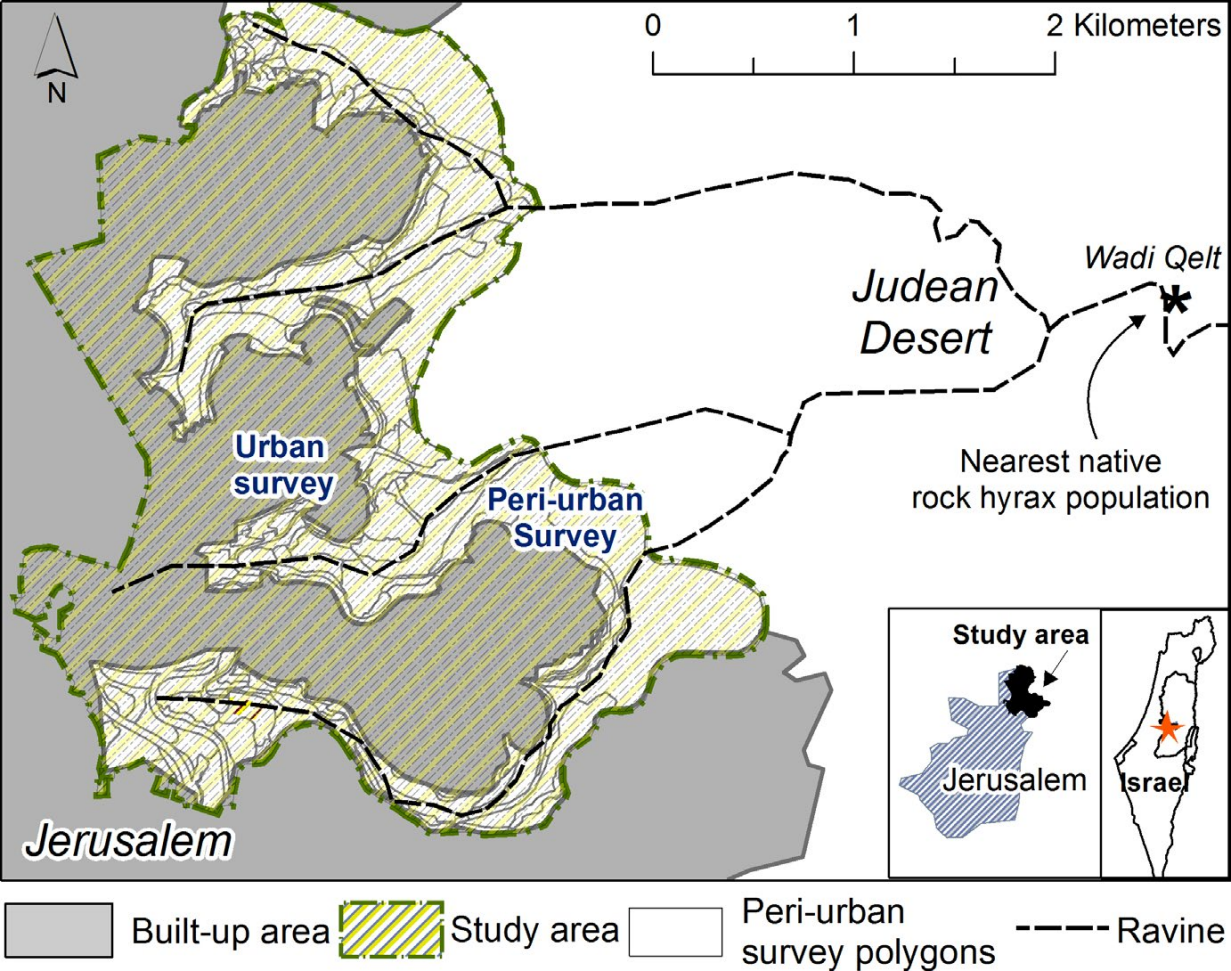
Map of the study area. The study area (dashed yellow lines) is located in the northeast of Jerusalem and comprises peri‐urban and core urban areas. To the east of the peri‐urban areas are the Judean Desert and the Wadi Qelt Nature Reserve where native rock hyrax populations reside

The study area is located in northeast Jerusalem, an extension of the city toward the Judean Desert (Figure 2). Here, the two neighborhoods of Pisgat Ze'ev and Neve Ya'akov (PZ and NY, respectively) were built during the 1980s on hilltops surrounded by dry ravines (wadis). These wadis join near the city and form Wadi Qelt, a natural canyon with a native rock hyrax population, located 4 km away from the study site.

Following the rural–urban approach, we divided the study site into the outer “peri‐urban” and the inner “urban” areas of the city (Figure 2). Unlike many other cities, where the boundaries of urban areas are hard to define as they merge into suburban development and also feature patches of rural areas (MacGregor‐Fors, 2010), in our study area these borders were clear as the outer and inner areas vary greatly from each other.

The “peri‐urban” area comprises a 2‐km‐wide belt of open land covering 4.16 km^2^, located at the interface between the wilderness of the Judean Desert and the Wadi Qelt Nature Reserve—an area with limited human presence and development, and the city (Figure 2). This area is devoid of any human residences but is littered with artificial rock piles created during the expansion of the city. It is mostly barren, with seasonal plant cover of grass and low shrubs.

The “urban” area covers 4.05 km^2^ and has the characteristics of an “urban core” (MacGregor‐Fors, 2010): It has high population density (20,000 residents per km^2^) and is mostly built (61% of the area) with residential blocks of 3–8 stories, wide roads, public buildings, and commerce. It contains two neighborhoods: PZ and NY (Figure 2), similarly designed in terms of city planning. In both neighborhoods, about 40% of the area comprises open spaces—private gardens, parks, or nonbuilt‐up areas, which can serve as foraging grounds for hyraxes. However, the two neighborhoods differ in their socio‐economic status and cultural norms, and these differences are reflected in the urban environment. PZ is the less conservative and more affluent neighborhood of the two and most of its public areas are well‐maintained. NY is poorer and hosts a large ultra‐Orthodox Jewish community, characterized by large and low‐income families (an average of 7.5 children per family – Kahaner, Malach, & Hoshen, 2017), residing in small apartments sometimes shared by multiple families. Lack of storage space in these households and a general disregard of environmental regulations have resulted in the accumulation of discarded furniture and other household items on open grounds, creating shelter opportunities for hyraxes. In addition, more shelters can be found under temporary structures (common in NY as semilegal building: Table A2). Moreover, the religious norms of the Orthodox Jewish residents of NY prohibit wasting edible food items, and thus, they tend to leave these exposed and available for the needed (Schwartz, 1997), as well as for animals.

### 2.2 Study species

The rock hyrax, *Procavia capensis*, is a medium‐sized (3–4 kg) social mammal found throughout the sub‐Sahara, North Africa and the Middle East (Butynski, Hoeck, Koren, & de Jong, 2015). While on the verge of extinction in the neighboring countries (Rifai, Baker, & Amr, 2000), in Israel, the species is regarded as protected (under the Law of Wildlife Protection, 1955), and common in a range of environments, from arid deserts to woodlands. Within its broad geographic range, the rock hyrax distribution is often patchy as its presence is tightly associated with the presence of rock piles or boulder concentrations (Barry & Mundy, 2002; Gerlach & Hoeck, 2001). Rock hyraxes rely on these rocky features for several reasons. They do not dig burrows and instead use natural rock crevices between rocks to rest and hide (Meltzer & Livneh, 1982) and always forage at a safe distance to return to these shelters (Druce et al., 2006). Possessing poor thermoregulation abilities, the rock hyraxes also rely on rocky shelters to escape from unfavorable weather conditions and solar radiation (Bartholomew & Rainy, 1971). They frequently control their body heat by basking to absorb heat from the rocks and move between shaded or exposed rocks according to the ambient temperature (Brown & Downs, 2007; Taylor & Sale, 1969). Rocks also used both as vantage points for sentinels, ensuring the safety of other group members foraging in nearby open grounds and as prominent spots for males to declare their territory (Meltzer & Livneh, 1982). Because of their significant dependency on rocky shelters, we focus here on shelter availability and quality as a main driver/limiting factor for their expansion.

### 2.3 Survey methods

We conducted the surveys during the summer months (July–August) of 2015, and again (only in the urban area) in July 2018. During these months, rock hyraxes are active in the early morning and before dusk and can be more easily spotted then (Meltzer & Livneh, 1982).

#### 2.3.1 Peri‐urban survey (2015)

**Figure 3:**
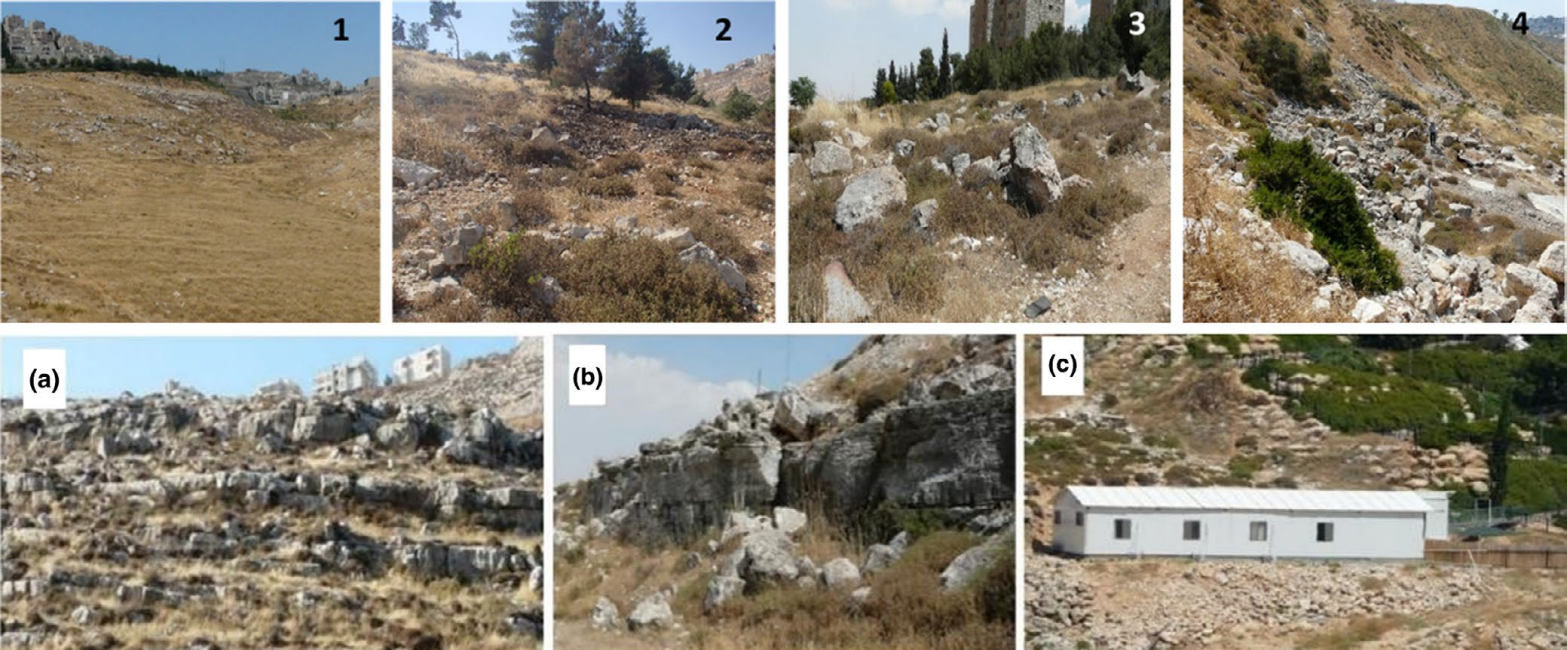
Examples of the classification of habitat units. Images 1–4 represent habitat classification according to “Quality” measurements: (1) An open area, without adequate refuge; (2) An area with few and scattered rocks that can serve for hidden movement or lookout; (3) An area with scattered rock mounds with crevices; (4) An area with continuous shelter options creating a crevice system with numerous openings. Images a–c represent classification according to “Type” characteristics: (a) Natural—cliffs and boulders formed by natural erosion. (b) Mixed (natural + artificial)—boulders that were pushed downhill from road construction and natural cliffs. (c) Artificial—portable structures with a wide crevice beneath, retaining walls, rock mounds

The survey was conducted on foot. First, we characterized the landscape according to refuge availability for *P. capensis*. We considered “shelter” as any rock formation (piles, retaining walls, and cliffs) with cavities large enough for hyraxes: deeper than 50 cm and with an opening diameter larger than 20 cm (based on our previous measurements in native hyrax habitats and on Meltzer & Livneh, 1982), we divided the peri‐urban area into 248 polygons (Figure 2) with each differing from its neighbors in its shelter characteristics (“quality” and “type”). We defined “quality” as a measure of availability and density of shelters on a scale of 1–4 (Figure 3):

(1) an open area without adequate refuge; (2) an area with few and scattered rocks that can serve for hidden movement or lookout; (3) an area with scattered rock mounds with crevices; (4) an area with continuous shelter options creating a crevice system with numerous openings. The shelter “type” was defined either as: (A) natural: cliffs and boulders formed by natural erosion; (B) artificial: any rock accumulation that was formed by human activity; or (C) mixed: a combination of rocky natural features with artificial forms (Figure 3a–c). We defined these characteristics on site and used high‐resolution (10 cm per pixel) georeferenced orthophotos (Survey of Israel, 2015) to accurately define the boundaries of each polygon.

We then surveyed the occurrences of *P. capensis* in each polygon during their peak activity hours (early mornings and late afternoons). We conducted a two‐stage observation: first, we surveyed each polygon from a distance of 50–100 m for 15 min. After observing hyraxes in a polygon, we approached and verified whether the animals had fled to shelter in their polygon or escaped to a neighboring one. Only polygons that sheltered hyraxes were considered “occupied”.

We estimated overall population size by examining hyrax abundance in 13 survey units that were colonized. These units were selected because they were of different sizes and because they were visible from two different vantage points. Counting was conducted in July 2015 by two independent observers from two different vantage points after sunrise (7–9 a.m.) and before sunset (5–7 p.m.), when the animals are most likely to be basking in the sun near their dens and easier to count. Rock hyrax colony sizes are often in correlation to the area of available shelter (Mbise et al., 2017). To estimate the colony sizes in the uncounted units according to the shelter size we conducted a curve fit test.

#### 2.3.2 Urban survey (2015)

We identified all the potentially “conventional” refuge sites for *P. capensis* (e.g., rock mounds and retaining walls) in the PZ and NY neighborhoods by analyzing high‐resolution orthophotos (10 cm per pixel) followed by ground surveys in all the streets, urban parks and open grounds. We characterized the urban sites using the shelter “quality,” “type,” and hyrax occurrence criteria we used in the peri‐urban area, but also defined the site's degree of maintenance as an indicator of the human attitude toward living environment.

We defined sites as of low maintenance effort (i.e., derelict) when they contained: (a) Temporary structures—an indication of unregulated building practices; (b) waste accumulation on open grounds; and (c) *Acacia saligna* thickets—an invasive bush that flourishes in disturbed soil and unkept gardens (Cohen & Bar, 2017). We considered sites as “maintained” where garbage is collected, and plants are trimmed and watered.

To locate urban hyrax colonies in unconventional shelters (i.e., sites without rocks, where hyraxes inhabited instead analogous refuges) or cryptic sites, we questioned over 100 residents (random passers‐by or by knocking on doors) in different parts of the neighborhoods. We showed them images of hyraxes and then asked whether they knew the locations of colonies and requested permission to access closed grounds (such as private gardens) when needed. We also met with police officers and neighborhood community administrators, who helped in providing additional information. When we found hyraxes colonizing unconventional sites, we categorized and detailed their habitat characteristics in the same manner as we had done for the conventional ones.

#### 2.3.3 Repeated urban survey (2018)

The successful establishment of hyrax colonies was examined by repeated observations during July 2018 (3 years after the first survey). We surveyed all colonized sites in 2015 and all vacant conventional sites during 2015, for the existence, disappearance, or new establishment of hyraxes at the sites. We also collected data obtained by the Jerusalem municipality and the Israel Nature and Parks Authority about other sightings of hyraxes in the neighborhoods and visited those sites to confirm hyrax colonization.

### 2.4 Statistical analysis

To determine the factors that facilitate hyrax colonization in the peri‐urban area, we constructed a model that examines the relationship between each polygon's characteristics and its colonization by hyraxes. The tested characteristics were as follows:

*Quality* and *Type* of shelters in the polygon (categorical values, as described earlier in the Methods section, Figure 3);

*Slope*, which measures the inclination of the topography in the polygon, based on ASTER digital elevation model (most natural populations occur in rugged topography);

*Proximity to urban area*, which is the distance from nearest residential structure (may attract due to food resources or deter due to human activity);

*Area*, log‐transformed size of the survey polygon (larger polygons may be more attractive as they may offer more shelters).

We performed a logistic regression using the following equation:

Y=Area+Quality+Type+Slope+Proximity to urban

Our sample data (*n = *248) were divided into training and test datasets. We selected randomly 75% of the samples as training data (*n = X*) and 25% as the test data (*n = Y*). The *glm* function from the statistical package *R* was applied with *binomial* family with *logit* link (R Core Team, 2013).

### 2.5 GIS analysis

An important attractor of hyraxes to human settlements is that of gardens and parks as foraging grounds (Kershenbaum & Blaustein, 2011; Moran et al., 1987). We estimated their area in the neighborhoods by analyzing orthophotos (10 cm per pixel) using the maximum likelihood classification tool in ESRI ArcGIS.

## 3 RESULTS

### 3.1 Hyrax colonies in peri‐urban areas

**Figure 4:**
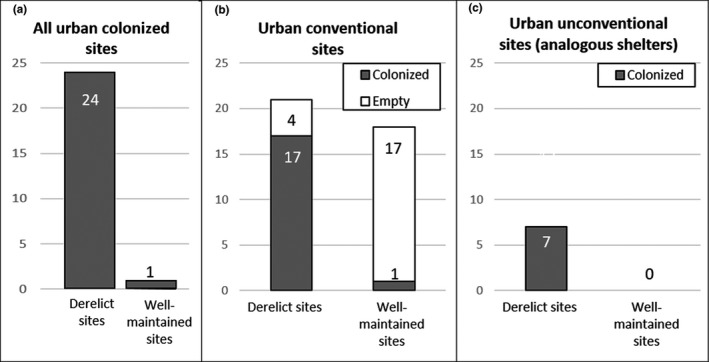
Rock hyrax colonization of urban sites according to derelict/maintenance: (a) in all the colonized urban sites; (b) only in conventional sites; (c) only in unconventional sites. Note that as unconventional shelters do not have typical form and can comprise numerous types of crevices, we surveyed only those that were colonized

The division of the peri‐urban area into 248 shelter units (polygons), according to their natural/anthropogenic origin and the quality of shelter, indicates that naturally the area is not suitable for hyraxes—it has no all high‐quality shelters (level 4) and only 2 shelters of level 3 (Table 1). We observed hyraxes in 47 of polygons (Figure 4), with a near‐total dependency (98% of all shelters) on artificial shelters. The shelters used were artificial rock mounds (86%), retaining walls (12%), and other one natural outcrop (2%).

**Table 1:** Refuge types for site units and the hyrax occurrences based on refuge quality index

Refuge type	Number of total units	Number of polygons *with Procavia capensis* (% of type colonized)	Number of polygons according to “refuge quality” index (% colonized)				total area (1,000 m^2^)	Mean unit area (1,000 m^2^)
			1	2	3	4		
Natural	39 (16%)	1 (2%)	18 (0%)	19 (5%)	2 (0%)	0	2,151	78
Mixed (natural + artificial)	38 (15%)	6 (16%)	7 (0%)	17 (0%)	5 (40%)	9 (44%)	699	21
Artificial	168 (69%)	40 (23%)	38 (3%)	14 (14%)	60 (10%)	56 (55%)	1,182	11

Note

Refuge types indicate if a unit (see Figure 2) is Natural, Artificial, or Mixed (combination of natural and artificial). Refuge quality indicates if the unit provides enough shelter with 1–4 index (see Figure 3).

The logistic regression confirmed a significant effect of the site characteristics on the occurrence of rock hyraxes. The final model after model selection was:

$$\text{Expected hyrax occurrence}={\text{Shelter}}_{\text{Quality}}+{\text{Shelter}}_{\text{Type}}+{\text{Shelter}}_{\text{Area}}$$

( $r^{2}=.92$ and AUC=0.803 ), confirming that shelters are indeed the main driver for the hyrax expansion to this area. *Shelter quality* is the most important predictor, with *shelter type* as well as shelter size also being statistically significant (Table 2a). Further analysis revealed that higher “shelter quality” contributes to the increased probability of hyrax occurrence; with “shelter quality” 4 having the highest effect. Area size also contributed to a higher probability of occurrence (Table 2b).

**Table 2a:** Results of logistic regression between the occurrence of *Procavia capensis* and the 248 polygons (shelter units) parameters—“type” (natural/man‐made/mixed), “quality” (on an ascending grade of 1–4), and “area size”

	*df*	Deviance	Resid.	*df* Resid.	Pr (>Chi)
NULL			182	189.61	
Unit type	2	6.907	180	182.7	0.032
Unit quality	3	47.559	177	135.14	<0.001
Unit area	1	4.069	176	131.07	0.044

Note

The results indicate the major effect of the shelter quality on hyrax occurrence and a lesser effect of its size.

**Table 2b:** Results of the logistic regression between the occurrence of *Procavia capensis* and the levels of shelter quality, indicating significant effect of high grades of shelter quality (3 and 4) on hyrax occurrence

	Estimate	*SE*	*z* value	Pr (≥*z*)
Intercept	−7.3904	2.0934	−3.530	>0.001
Type (mixed artificial + natural)	−0.6283	0.6537	−0.961	0.336
Type (natural)	−0.3906	1.0727	−0.364	0.716
Quality level 2	0.9721	1.2643	0.769	0.442
Quality level 3	2.5951	1.1543	2.248	0.025
Quality level 4	4.3831	1.1075	3.958	>0.001
Unit area	0.3972	0.2011	1.975	0.048

When comparing shelter characteristics and occupancy between the peri‐urban area adjacent to the PZ and the NY neighborhoods, we found that the high‐quality shelters (level 4) near NY were more common (25% of the total shelters compared to 8% around PZ; Figure 4), and were generally larger in size (average 9,700 m^2^ compared to 4,300 m^2^ in PZ). Moreover, most of the high‐quality shelters (87%) near NY were occupied, leaving only limited options for further expansion, compared with 47% in the peri‐urban areas around PZ. These findings that high‐quality shelters are more common and more saturated with hyraxes located near the PZ neighborhood, may indicate a higher potential for further expansion to the urban areas of that neighborhood.

**Figure A1:**
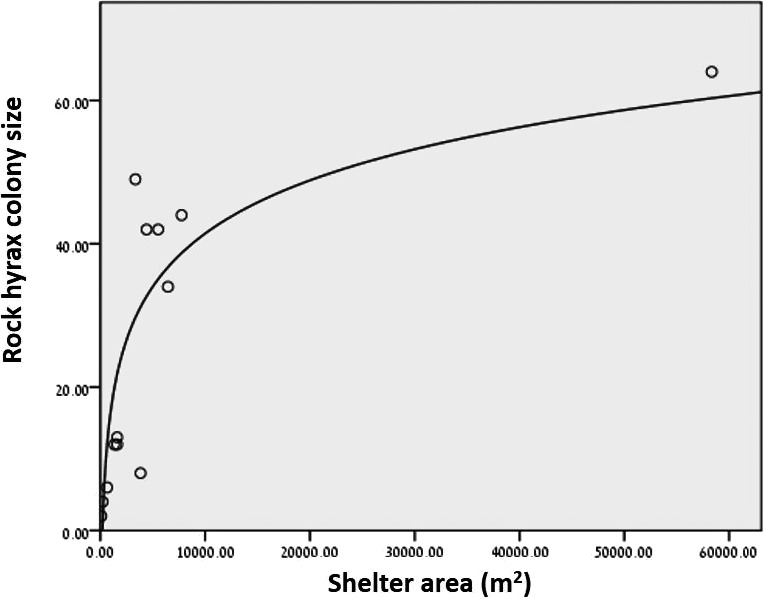
Rock hyrax colony size as function of shelter area in the peri‐urban region. The curve fit represents the logistic equation: Hyrax colony size = 10.71 ln (area_(m)_) − 57.13 $r^{2}=.92$ and ( AUC=0.803 ), We did not refer to the extreme point at the right side of the graph as an outlier, because the numbers of hyraxes in a colony are limited due to their social structure, and In Israel, they typically do not exceed 65 individuals (Meltzer & Livneh, 1982)

Most of the high‐quality shelters were found in close proximity to the urban area and human residences (63%) <50 m, while only 7% were >150 m from these (Table 2a and 2b). The peri‐urban hyraxes seemed to take advantage of their close proximity to urban foraging grounds: We frequently spotted hyraxes foraging in parks and gardens in the urban periphery and retreating to peri‐urban shelters when disturbed (Figure A1). We identified 11 such peri‐urban colonies that forage in urban parks, but the actual number may have been higher.

**Figure A2:**
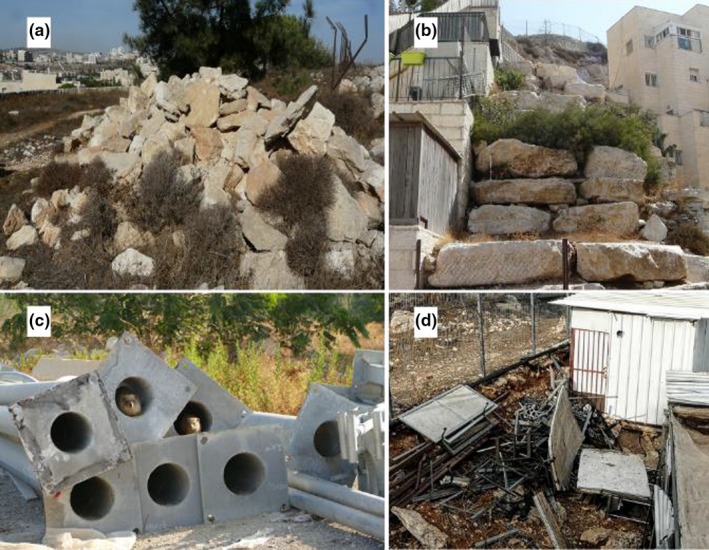
Examples of urban sites with hyrax colonies. a and b represent “conventional” rock shelters (rock mounds and retaining walls); c and d represent “unconventional” shelters—piles of construction materials and dry waste in derelict grounds

We found a significant relationship between the shelter area and hyrax colony size:

Hyrax colony size = 10.71 X ln (area_(m)_) − 57.13 ( $r^{2}=.92$ and AUC=0.803 ), (Figure A2). Based on this equation we estimated the total peri‐urban population size as 1,600 individuals, distributed evenly between the peri‐urban areas of the two neighborhoods.

### 3.2 Hyraxes in urban areas

**Figure 5:**
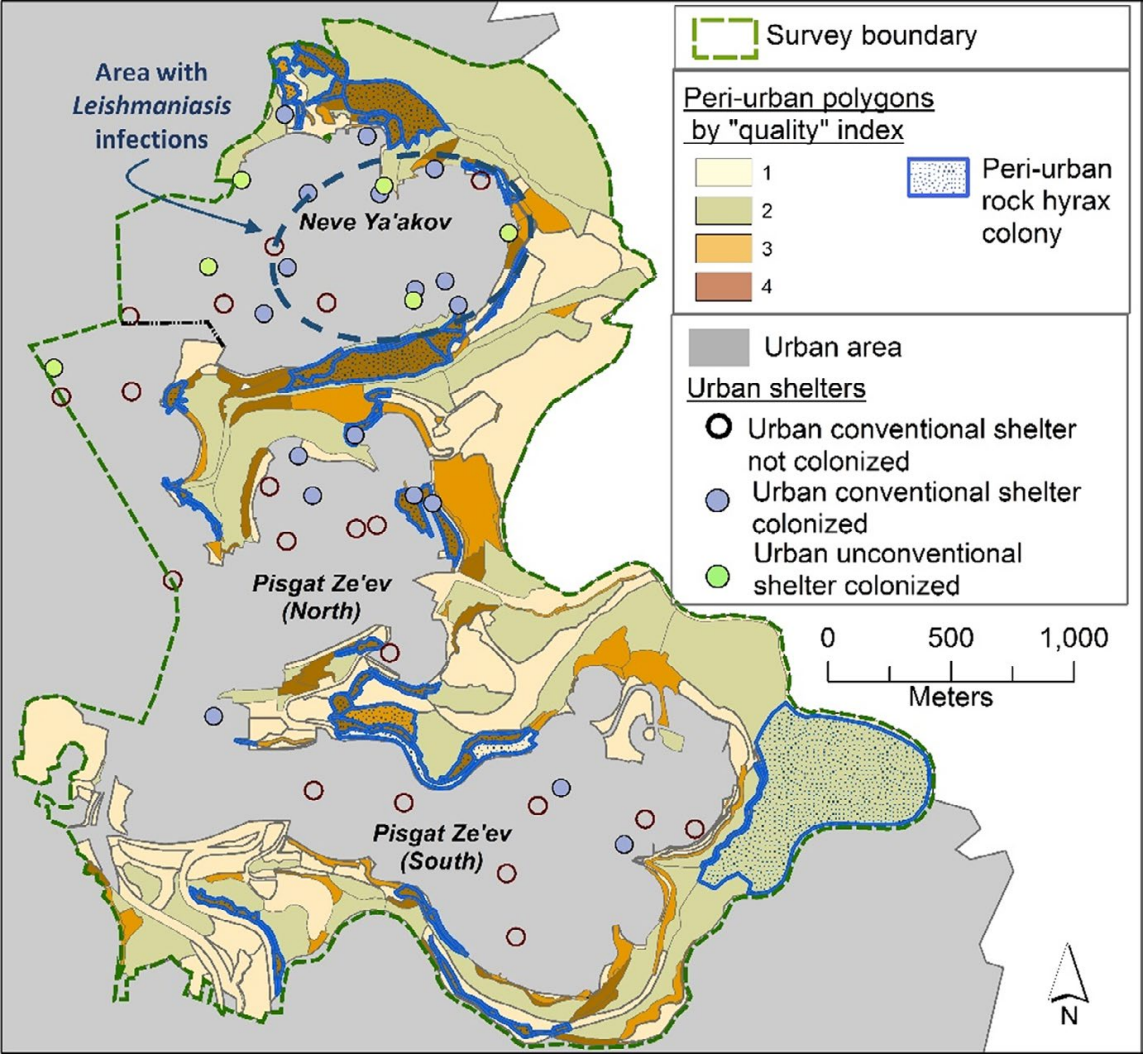
The evaluation of habitat quality and the locations of hyrax colonies in the peri‐urban and urban areas. Most of the high‐quality shelters of the peri‐urban area (dark brown polygons) as well as hyrax colonies (polygons with blue outlines) are concentrated at the fringes of the built (urban) area. The majority of urban hyrax colonies are concentrated in the Neve Ya'akov neighborhood where they occupy both conventional and unconventional (analogous) shelters and where leishmaniasis outbreak has occurred

Inside the urban areas, we located 25 permanent colonies (and Figures 4 and 5) with a significant preference to settle in derelict sites. Overall, we found only one hyrax colony in the 14 well‐maintained urban sites that had high‐quality shelters (rock mounds and retaining walls), compared with 24 urban colonies at derelict sites.

In terms of availability of urban shelter sites, we located 38 sites featuring “conventional” high‐quality rocky shelters (refuge quality = 4, Figure 5): 16 in NY and 22 in PZ. These were mostly retaining walls that resemble rock piles with deep crevices. Among these conventional sites, we found hyrax colonies in 18 of them (47%): 10 in NY and 8 in PZ (Figure 5).

Rock hyrax colonies were more common in the NY neighborhood—18 colonies compared with seven in PZ, despite NY being half the size of PZ. While all the colonies in PZ resided in conventional shelters, in MY we located seven colonies in sites that contained only “unconventional” shelters and are features unique to this neighborhood: cavities under temporary structures (part of an unauthorized building activity), and inside dumpsites formed by residents in public grounds (Figure A1).

**Figure A3:**
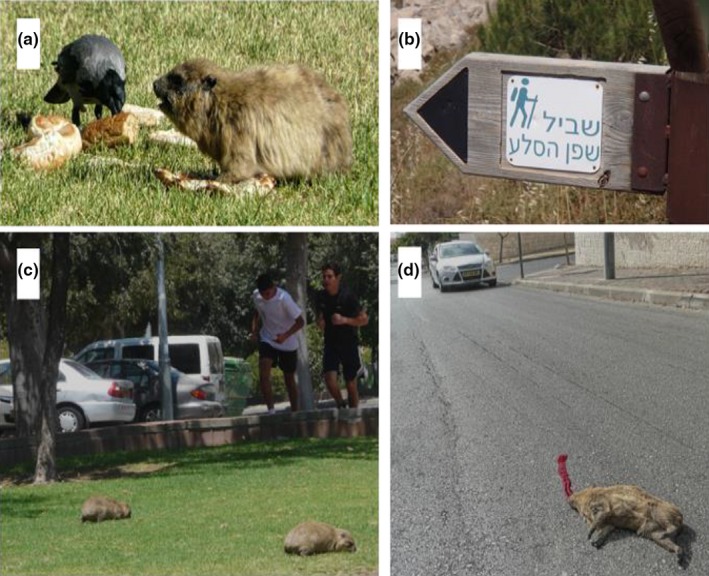
Examples of hyrax life in the city: (a) Food leftovers not disposed in dustbins due to cultural norms prohibiting wasting food are common features in religious parts of Neve Ya'akov neighborhood, serving as an additional food source for urban hyraxes and hooded crows; (b) Hyrax observation trail designed by the municipality demonstrating the tolerance toward the new invaders before recognized as pests; (c) Urban hyraxes and joggers share grounds in a city park; (d) Hyraxes adapting to the urban region face new risks like crossing roads

In terms of differences in foraging availability between the neighborhoods, remote sensing analysis for foraging grounds revealed that green open spaces (i.e., private gardens, parks or other open areas with plant cover) comprise a similar percentage of the area in both neighborhoods (approximately 40%). Moreover, the total area of green foraging ground is actually much larger in PZ (total area of 1.4 km^2^ and 0.4 km^2^ in PZ and NY, respectively). However, NY contains “unconventional” forage options in terms of food scraps from residents' homes that are dumped on public grounds, driven by of religious rules that prohibit throwing food to the garbage, and lack of awareness by the local community. We observed hyraxes feeding on such human waste in 8 sites in NY (Figure A3).

Reconnaissance surveys in 2015 and 2018 indicated that the hyrax colonies in the core urban area of this study have become established and reproduce. Out of the 25 colonies observed in 2015, we confirmed 18 of these colonies again in 2018. Of the seven colonies that had disappeared, four sites had been demolished due to the construction of new buildings or other infrastructure. We also observed that one formerly empty site was now occupied by a new colony including juveniles. However, this colony might have moved from an adjacent, formerly occupied site that was found empty in 2018. Overall, we observed juveniles in eight colonies in 2018, of which seven had existed since 2015.

We further confirmed new observations obtained from the Jerusalem municipality and Israel Nature and Parks Authority and located five more colonies, to the west of PZ and NY neighborhoods. All the colonies inhabit artificial rock mounds at a peri‐urban area and are less than 100 m from the urban area. Their location, to the west of the former urban colonies which we located in 2015, and further away from the native range , suggests that the urban hyraxes disperse through the city in a leap‐frog pattern (Evans et al., 2010) and used a bottleneck of the urban area to colonize favorable sites with the combination of undisturbed shelters and close urban forage.

## 4 DISCUSSION

In this study, we analyze urban expansion of rock hyraxes into Jerusalem along a rural–urban gradient, by focusing on shelter availability. Our results indicate that land cover changes of the peri‐urban areas have promoted hyrax expansion through the creation of new rocky habitats, while the socio‐economic conditions and cultural norms of human residents are providing niches in the core urban areas where hyraxes find undisturbed shelters and supplementary food. Additionally, we document for the first time that rock hyraxes have become established in the densely populated urban areas, and their populations in core urban areas are reproducing.

Our results indicate that the peri‐urban area of Jerusalem lacks high‐quality natural refugia in terms of shelter availability (Table 1). Instead, we found that the hyraxes have populated this area by using new artificial refuge opportunities formed by road and building construction related to the urban development (Table 1). We demonstrated that this colonization of the peri‐urban area has a predictable pattern (Table 3), with most high‐quality artificial shelters having become occupied (Table 2a and 2b), and concentrated along the edges of the urban area (Table 4).

**Table 3:** Potential drivers for shifts from the peri‐urban areas to the adjacent urban areas

Parameter measured	Peri‐urban of Neve Ya'akov	Peri‐urban of Pisgat Ze'ev	implication
Estimated rock hyrax population size	770	810	High densities compared to natural populations
Occupancy of suitable habitats (quality 3–4)	87%	47%	Near saturation of suitable habitats in Neve Ya'akov
Number of rock hyraxes per 1 km of neighborhood perimeter bordering peri‐urban area	250	70	Potential higher invasion pressure from Neve Ya'akov compared to Pisgat Ze'ev
% of Neighborhood perieter bordering peri‐urban colonies	63	37	

**Table 4:** Distances of high‐quality habitats (grades 3–4) and hyrax colonies from residential areas

Distance from edge of residential area (m)	% of the total area of high habitat quality (grades 3–4)	% units with hyrax colonies	% of area with high habitat quality (grades 3–4)
0–50	70.20	63	29
50–150	23.40	30	17
>150	6.40	7	12

Note

More than 90% of the high‐quality habitats and most of the rock hyrax colonies in the peri‐urban areas are concentrated at close proximity (<150 m) from the residential areas.

Our observations support previous studies (Mbise et al., 2017; Wiid & Butler, 2015) that rock hyraxes benefit from living close to human settlements, likely due to the readily available food resources and lack of natural predators. In the peri‐urban areas, we found that their numbers are much higher than native populations in the nearby Judean Desert; 1,600 individuals compared with 200 in the Arugot Nature Reserve, an oasis of similar size with abundant shelters and vegetation (Geffen E.*,* Tel Aviv University, personal communication, 2019). High densities in hyrax populations are known to increase competition and aggression (Gerlach & Hoeck, 2001; Hoeck, 1989), which push young animals lower down in the hierarchy into exile (Koren, Mokady, & Geffen, 2006; Meltzer & Livneh, 1982), or into human settlements (Wiid & Butler, 2015). As the potential man‐made habitats around the Neve Ya'akov neighborhood have become exhausted (Table 3), such saturation of resources likely creates higher pressure for hyrax colonies to spill over into the adjacent core urban areas.

In addition to these external drivers from the peri‐urban area, internal attractive conditions in the urban area may also contribute to the hyrax invasion to the city. Specifically, we found that in the Neve Ya'akov neighborhood hyraxes take advantage of food and shelter alternatives formed by the local socio‐economic conditions and cultural norms (Table A2); they use dry waste accumulated in public spaces for shelter as well as crevices under temporary structures, which are common features of illegal building activity the in poor neighborhoods of Jerusalem. We observed that they also forage on exposed human food leftovers, which are not disposed in dustbins due to cultural norms prohibiting food waste (Figure A3). Trash accumulated in urban areas is known to attract other wildlife species to cities, including spotted hyenas in Ethiopia (Yirga et al., 2015), white storks in Poland (Kruszyk & Ciach, 2010), coyotes in Canada (Murray et al., 2016) and golden jackals in Israel (Borkowski, Zalewski, & Manor, 2011). In both neighborhoods, hyraxes avoided conventional shelters in well‐maintained areas where they were likely persecuted by maintenance workers or gardeners (Table A2).

While an underlying proximate cause of rock hyrax invasion is human‐related, the intrinsic nature of the rock hyrax may also play a role; one factor is its habituation to human presence, and another its exploitation of novel habitats (Naylor, 2015). In contrast to some other studies on urban wildlife that involved temporal and spatial segregation from a human presence (Ditchkoff et al., 2006; Gaynor, Hojnowski, Carter, & Brashares, 2018), the hyraxes in Jerusalem show little fear of humans. They often dwell inside public buildings and are active next to human presence (e.g., crossing roads with traffic; Figure A2). Such behavioral adjustment may also facilitate the species’ urban invasion (Blumstein, 2014). As seen in other urban animals such as stone martens (Herr, Schley, Engel, & Roper, 2010), large‐spotted‐genets (Widdows, Ramesh, & Downs, 2015) and raccoons (Hadidian, Prange, Rosatte, Riley, & Gehrt, 2010), the rock hyraxes in Jerusalem exploit diverse urban structures as analogues to their natural shelters, suggesting that they are highly adaptable.

Our results did not show how well the urban analogues to rocky shelters function as refuge from cold in winters. This may pose a challenge for the rock hyrax colonization considering that Jerusalem is located at an elevation of >650 m in mountain terrain, while the native hyrax population resides in lower elevations of <300 m within secluded canyons. However, the environmental conditions that prevail in the city, such as higher ambient temperatures in dense urban areas during winters (Pickett et al., 2001), may compensate for the inferior insulation characteristics. Moreover, the urban colonies enjoy reliable food sources throughout the year and are not limited by seasonal fluctuations in foraging, unlike the native desert populations.

Our results indicate that the rock hyraxes have successfully expanded their distribution into the city of Jerusalem and are now adjusted to the urban environment. Evans et al. (2010) have suggested that the wildlife urban expansion occurs in three stages: (a) arrival in urban areas; (b) adjustment to the urban environment; and (c) spread within urban areas and into neighboring towns and cities. The further dispersal and colonization into more densely populated areas of the city have considerable implications for the exposure of humans to leishmaniasis outbreaks (Salah, Abbasi, Warburg, Davidovitch, & Kotler, 2020; Salah, Davidovitch & Kotler, 2016). Identification of the drivers that promote the wildlife urban expansion, such as relevant land cover changes, human cultural norms, and areas of municipal neglect, are necessary for recognizing more areas at risk of colonization and implementing appropriate and relevant prevention measures.

## CONFLICT OF INTEREST

The authors certify that they have NO affiliations with or involvement in any organization or entity with any financial interest, or nonfinancial interest in the subject matter or materials discussed in this manuscript.

## AUTHOR CONTRIBUTIONS

**Noam Ben‐Moshe:** Conceptualization (equal); Data curation (lead); Formal analysis (equal); Investigation (lead); Methodology (equal); Validation (equal); Visualization (equal); Writing‐original draft (equal). **Takuya Iwamura:** Conceptualization (equal); Data curation (supporting); Formal analysis (equal); Investigation (supporting); Methodology (equal); Validation (equal); Visualization (equal); Writing‐original draft (equal); Writing‐review & editing (equal).

## Data Availability

We agree to archive the data associated with this manuscript should the manuscript be accepted. Data used to produce the analyses and figures in this manuscript will be archived in the Dryad Repository: https://doi.org/10.5061/dryad.73n5tb2t2

## APPENDIX 1

**Table A1:** Peri‐urban survey—data for the shelter units (polygons)

Naturality: 1‐ artificial; 2‐ mixed; 3‐ natural
Quality: 1‐ no available shelter; 2‐ scattered rocks without crevices; 3‐ scattered rock mounds with crevices; 4‐ continuous shelter, a crevice system with numerous openings.
Hyrax occurrence: 0‐ not observed; 1‐ observed
Urban proximity: 1‐ <50 m; 2‐ 50−150 m; 3‐ >150 m

Polygon Number	Naturality	Quality	Hyrax occurrence	Urban proximity	Area (sqm)	Description
1	1	3	1	1	645	Rock mound
2	2	2	0	1	13,966	Natural rock face with crevices
3	1	1	0	2	2,327	Construction working plot
4	1	3	1	2	4,409	Rock mound
5	1	3	1	3	3,922	Rock mound
6	1	3	0	1	1,487	Rock mound
7	1	3	0	1	3,165	Rock mound
8	2	2	0	1	1,781	Dirt road
9	1	4	1	2	5,317	Rock mound
10	1	4	0	2	4,737	Rock mound
11	2	4	0	2	1,325	natural rock face with crevices + Rock mound
12	1	4	0	1	2,032	Rock mound
13	1	3	0	1	628	Retaining wall
14	1	4	1	1	3,344	Big boulders pile
15	2	4	1	1	6,146	Rock mound
17	3	4	1	2	54,302	natural rock face with crevices + Big boulders pile
18	2	2	0	1	12,471	Sparsely scattered rock mounds
19	1	3	0	1	306	Retaining wall
20	2	2	0	1	12,253	Scattered boulders
21	1	3	0	1	2,668	Scattered rocks
22	1	4	0	1	785	Big boulders pile
23	2	4	0	1	7,255	Big boulders pile
24	1	2	0	1	2,345	Rock and dirt mounds
25	2	4	1	1	1,349	Big boulders pile
26	1	4	0	1	4,145	Rock mound
27	1	2	1	1	5,380	Rock and dirt mounds
28	3	1	0	1	4,528	Open area without rocks
29	3	2	0	2	338,079	natural rock face with crevices
30	1	3	0	1	1,800	Rock mound and dirt
31	3	3	0	2	18,422	natural rock face with crevices
32	1	3	0	1	2,536	Rock mound
33	1	3	0	1	4,889	Rock mounds
34	1	3	0	1	543	Retaining wall
35	1	4	0	1	5,023	Rock mound
36	1	1	0	1	9,226	Dirt mound
37	1	4	1	1	4,251	Rock mound
38	1	3	0	2	2,123	Rock mound
39	1	4	1	1	6,031	Rock mound
40	3	1	0	2	36,355	Flat terrain without rocks
42	3	1	0	2	76,840	Natural rocky ground
43	2	2	0	1	73,376	Scattered small rock mounds
44	3	3	0	3	3,884	Natural rock mounds
45	3	2	0	2	58,964	Scattered natural rock mounds
46	3	1	0	1	199,366	Grass area without rocks
47	1	3	0	1	741	Rock mounds and dirt road
48	2	2	0	1	23,351	Scattered boulders
49	2	4	1	1	58,335	Big boulders pile
50	1	4	1	1	4,319	Rock mounds
51	2	2	0	1	13,013	Dirt mound
52	1	4	1	1	2,683	Rock mounds
53	1	4	1	1	1,566	Rock mounds
54	1	3	0	1	9,226	Rock mound
55	2	3	0	2	35,850	Scattered boulders
56	1	4	1	1	5,247	Rock mound
57	1	4	1	1	10,146	Rock mounds
58	1	4	0	1	11,942	Rock mound and retaining wall
59	1	1	0	1	850	Old Quarry
60	1	4	0	2	11,834	Big boulders pile
61	1	4	0	2	13,235	Rock mounds
62	3	2	0	1	111,149	Scattered boulders
63	1	4	1	1	3,613	Retaining wall
64	1	4		1	1,039	Retaining wall
65	1	3	0	1	3,003	Rock mounds
66	1	4	1	1	1,618	Rock mounds
67	1	4	1	1	1,627	Retaining wall
68	1	3	0	1	5,249	Rock mound
69	1	4	0	1	12,971	Rock mound
70	1	3	0	1	2,266	Small retaining wall
71	1	4	1	1	5,621	Rock mound
72	1	4	1	1	714	Rock mound
73	1	1	0	1	2,756	Dirt mound
74	1	4	0	1	2,804	Dirt mound with scattered rocks
75	3	2	0	1	40,158	Scattered boulders
76	3	2	0	1	10,989	Scattered boulders
77	1	2	0	2	31,457	Rock mounds inside construction working plot, dry waste
78	1	4	0	2	8,903	Rock mound
79	1	3	0	1	4,855	Rock mound
80	1	3	0	1	360	Rock mound
81	1	3	0	3	4,450	Rock mound
82	1	3	0	1	782	Rock mound
83	1	3	0	1	70,278	Rock mound
84	1	3	1	1	217	Retaining wall
85	1	4	1	1	11,568	Rock mound
86	1	3	1	1	95	Retaining wall
87	1	4	1	1	13,334	Rock mound
88	1	3	0	1	512	Retaining wall
89	1	3	0	1	301	Retaining wall
90	1	2	0	1	15,608	Construction working plot with scattered rocks
91	1	3	0	1	264	Retaining wall
92	1	3	1	1	2,544	Rock mound
93	1	4	1	3	7,753	Rock mound
94	2	1	0	2	5,810	Open area without rocks
95	1	2	0	2	18,892	Dirt slope
96	1	4	1	3	12,028	Old Quarry
97	2	1	0	1	3,042	Open area without rocks
98	2	1	0	1	15,821	Open area without rocks
99	3	2	0	2	6,346	natural rock face with crevices and scattered rocks
100	3	2	0	1	15,754	Forested slope
101	3	2	0	2	32,382	natural rock face with crevices in a ravine
102	3	1	0	3	25,055	Open area without rocks
103	1	4	1	1	9,770	Big boulders pile
104	1	1	0	1	49,335	Built with concrete
105	2	1	0	1	9,935	Open area without rocks
106	1	4	0	1	189	Retaining wall
107	1	1	0	1	4,087	Built with concrete
108	1	1	0	1	6,925	Built with concrete
110	1	1	0	1	4,156	Disturbed area between houses
111	1	4	1	1	5,045	Boulder mound
112	2	2	0	2	9,011	Disturbed area with small cliffs
113	2	4	0	1	12,341	Rock mounds in slope
114	2	4	0	1	7,031	Rock mound
115	2	2	0	1	3,165	Open area without rocks
116	1	3	0	1	15	Retaining wall
117	1	2	0	1	12,999	Disturbed area with small mounds
118	1	2	1	1	498	Small retaining wall
119	3	1	0	1	11,634	Open area without rocks
120	1	3	0	1	3,352	Rock mounds under road
121	1	1	0	1	3,343	Open area without rocks
122	2	1	0	1	7,239	Open area without rocks
123	3	1	0	2	16,002	Open area without rocks
124	1	4	1	2	5,525	Retaining wall
125	1	4	1	2	4,364	Boulder mounds
126	1	1	0	2	10,899	Open area without rocks
127	1	1	0	1	2,758	Orchard
128	1	1	0	1	2,260	Built with concrete
129	3	1	0	1	25,271	Open area without rocks
130	1	4	1	1	6,911	Boulder mounds under houses
131	1	1	0	1	1,634	Dirt mound
132	1	1	1	1	31,960	Dirt mound
133	1	4	1	1	11,856	Rock mounds
134	2	3	1	2	23,311	Natural rock faces and scattered rocks
135	3	2	0	2	29,113	Natural rock faces and scattered rocks
136	1	4	1	1	6,451	Boulder mounds under houses
137	1	4	1	2	8,704	Boulder mounds under houses
138	3	2	0	2	43,263	Natural rock faces
139	1	3	0	1	3,473	Rock mound under road
140	1	4	0	1	8,680	Rock mound between retaining walls
141	3	1	0	1	49,486	Open area without rocks
142	2	2	0	1	20,303	Disturbed area with scattered rocks
143	3	1	0	2	85,144	Open area without rocks
144	1	4	0	1	481	Retaining wall under houses
145	1	3	0	1	2,990	Rock mound under road
146	1	3	0	3	2,280	Rock mound under road
147	3	2	0	3	60,838	Natural rock faces
148	1	3	0	3	3,160	Rock mound under road
149	1	2	0	1	90,849	Natural rock faces
150	1	3	0	1	20,597	Old quarry with scattered rock mounds
151	1	3	0	1	2,120	Rock mounds
152	1	1	0	1	36,434	Disturbed area between houses
153	1	2	0	1	5,993	Disturbed area between houses
154	3	2	0	2	163,318	Natural rock faces
155	1	3	0	1	28,895	Disturbed area with scattered rock mounds
156	3	2	1	3	304,476	Natural rock faces
157	2	3	0	2	2,207	Rocky slope with scattered rock mounds
158	1	3	0	1	317	Retaining wall under road
159	1	3	0	1	9,283	Rock mound under road
160	1	1	0	1	3,811	Open area without rocks
161	1	3	0	1	11,330	Rock mound under road
162	1	4	0	3	1,435	Retaining wall
163	1	3	0	3	255	Retaining wall
164	1	3	0	3	544	Retaining wall
165	1	4	0	2	4,413	Rock mound under road
166	1	4	0	1	10,099	Rock mound under road
167	1	3	0	1	380	Retaining wall under road
168	1	4	0	1	950	Retaining wall under road
169	2	2	0	2	60,146	Rocky slope with scattered rock mounds
170	1	3	0	3	15,083	Rocky slope with scattered rock mounds
171	1	1	0	3	15,123	Dirt mound
172	1	3	0	3	101	Retaining wall
173	1	2	0	3	1,353	Scattered rocks
174	1	1	0	1	12,461	Orchard
175	1	1	0	1	1,777	Orchard
176	1	1	0	1	2,959	Orchard
177	1	1	0	1	3,952	Dirt mound
178	3	1	0	2	2,535	Open area without rocks
179	2	1	0	1	85,315	Open area without rocks
180	1	4	0	3	6,805	Rock mound under road
181	2	3	1	2	6,905	Rock mounds in a ravine
182	3	2	0	1	29,762	Rock mounds in a ravine
183	1	3	0	1	981	Retaining wall under road
184	1	4	1	2	13,406	Rock mound under road
185	1	1	0	2	13,670	Dirt mound
186	3	1	0	2	26,174	Open area without rocks
187	2	2	0	1	41,437	Scattered rocks in forested slope
188	2	4	0	1	5,538	Rock mound under road
189	1	4	0	2	1,374	Retaining wall under road
190	1	4	0	2	1,179	Retaining wall under road
191	1	4	1	1	1,651	Retaining wall under road
192	1	4	1	1	3,834	Boulder mounds
193	2	2	0	2	10,872	Natural rock faces
194	1	4	0	2	3,159	Rock mound under road
195	1	4	1	1	5,172	Rock mound under road
196	1	1	0	1	3,587	Construction working plot
197	1	3	0	1	4,684	Dry waste piles
198	1	2	0	1	12,753	Rock mound in a ravine
199	1	3	0	2	3,402	Rock mound
200	1	3	0	1	377	Retaining wall
201	1	3	0	1	5,450	Dirt mound
202	1	1	0	1	6,888	Construction working plot
203	3	1	0	2	6,141	Open area without rocks
204	1	3	0	1	5,572	Rock mounds
205	3	2	0	2	17,664	Open area with scattered rocks
206	1	1	0	1	6,358	Dirt mound
207	1	2	0	2	7,066	Rock mound in a ravine
208	1	1	0	1	2,736	Open area without rocks
209	1	4	0	1	1,215	Rock mound
210	3	1	0	1	6,774	Open area without rocks
211	1	1	0	2	21,193	Dirt mound
212	1	4	1	2	14,917	Rock mound beneath road
213	3	2	0	1	31,325	Rock mounds
214	1	3	0	3	3,676	Scattered rocks
215	2	2	0	2	24,324	Slope with scattered rocks
216	1	4	0	1	4,028	Rock mound under buildings
217	1	1	0	1	16,726	Dirt mounds
218	3	2	0	2	13,501	Natural rock faces
219	1	1	0	2	3,599	Dirt mound
220	3	1	0	2	18,703	Orchard
221	2	2	0	3	19,317	Retaining wall
222	3	1	0	3	23,313	Open area without rocks
223	1	3	0	1	5,560	Rock mound beneath road
224	2	3	0	1	7,106	Big scattered rocks
225	2	2	0	1	44,770	Slope with terraces
226	2	1	0	1	10,867	Open area without rocks
227	1	1	0	1	12,880	Road
228	1	1	0	1	26,630	Open area without rocks
229	3	1	0	1	6,170	Open area without rocks
230	1	1	0	1	6,459	Orchard
231	1	1	0	2	3,954	Orchard
232	1	3	0	1	1,024	Rock mound
233	1	1	0	1	2,650	Built with concrete
234	1	1	0	1	5,024	Orchard
235	1	1	0	1	1,873	Built with concrete
236	1	3	0	1	8,755	Rock mound
237	1	2	0	1	316	Retaining wall
238	1	2	0	1	1,341	Retaining wall
239	1	3	0	1	3,151	Rock mound
240	1	3	0	1	1,038	Retaining wall
241	1	3	0	1	2,348	Retaining wall
242	1	3	0	1	675	Retaining wall
243	3	2	0	2	26,249	Slope with natural rock faces
244	3	2	0	2	20,591	Scattered rocks
245	2	2	0	1	3,162	Scattered rocks
246	3	1	0	2	678	Road
247	1	1	0	1	19,004	Disturbed area next to construction site
248	1	1	0	1	6,528	Dirt mound

**Table A2:** Urban survey –Habitat features of the urban sites colonized by hyraxes. Gray columns contain characteristics associated with derelict areas and unregulated construction

Neighborhood	Site number	Conventional		Unconventional			Maintainenece	Occurrence in 2015 Survey	Occurrence in 2018 Survey
		Retaining wall	Rock piles	Mobile structures	Waste accumulation	*Acacia saligna* thickets		✓ = occurred − = absent	✓ = occurred; − = absent; O = site does not exist in 2018
Neve Ya'akov	255			+			−	✓	✓
	256			+		+	−	✓	✓
	258	+				+	−	✓	✓
	259	+				+	−	−	−
	261	+				+	−	✓	O
	262	+					+	−	−
	263	+		+	+		−	✓	✓
	264	+		+	+		−	✓	✓
	265	+			+		−	✓	✓
	266			+	+	+	−	✓	✓
	267			+	+		−	−	−
	268	+			+	+	−	✓	✓
	269	+			+		−	✓	✓
	270	+		+		+	−	✓	✓
	271	+					+	−	−
	273			+	+		−	✓	✓
	275	+			+		−	✓	O
	276	+			+		−	✓	✓
	278	+					−	−	−
	279	+					−	−	−
	280	+					+	−	−
	297				+		−	✓	✓
	323			+			−	✓	✓

Neighborhood	Site number	Conventional		Unconventional			Maintainenece	Occurrence in 2015 Survey	Occurrence in 2018 Survey
		Retaining wall	Rock piles	Mobile structures	Waste accumulation	*Acacia saligna* thickets		√ = occurred X = absent	√ = occurred; X = absent; O = site does not exist in 2018
Pisgat Ze'ev	284	+	+		+		−	√	X (hyrax found in nearby site that was previously unoccupied)
	287	+					+	X	X
	288	+					+	X	X
	289	+					+	X	X
	290	+			+	+	−	X	X
	293	+					+	X	X
	294	+					+	X	X
	296	+					+	X	X
	298	+					+	X	X
	301	+				+	−	X	X
	302	+					+	√	√
	305		+				−	√	O
	309	+					−	√	X
	310		+			+	−	X	X
	311	+					+	X	X
	312	+					+	X	X
	314	+					+	X	X
	317		+				−	√	O
	318		+				−	√	O
	320	+	+				−	√	√
	321		+				−	√	√
	323		+				−	X	√

Note

Right column shows occupancy of sites 3 years after the first survey.
